# Social-economic analysis of patients with Sjogren’s syndrome dry eye in East China: a cross-sectional study

**DOI:** 10.1186/s12886-018-0694-5

**Published:** 2018-02-02

**Authors:** Wang Yao, Qihua Le

**Affiliations:** Department of Ophthalmology, Eye & ENT Hospital of Fudan University, No.83 Fenyang Road, Shanghai, 200031 China

**Keywords:** Sjogren’s syndrome, Dry eye, Medical expenditure, Psychological status, East China

## Abstract

**Background:**

Sjogren’s syndrome is the leading cause for aqueous tear-deficiency dry eye. Little is known regarding the relationship between Sjogren’s syndrome dry eye (SSDE) and patients’ medical expenditure, clinical severity and psychological status changes.

**Methods:**

Thirty-four SSDE patients and thirty non-Sjogren’s syndrome dry eye (non-SSDE) subjects were enrolled. They were required to complete three self-report questionnaires: Ocular Surface Disease Index, Zung Self Rating Anxiety Scales, and a questionnaire designed by the researchers to study the patients’ treatment, medical expenditure and income. The correlations between expenditures and these parameters were analyzed.

**Results:**

The annual total expenditure on the treatment of SSDE was Chinese Yuan 7637.2 (approximately US$1173.8) on average, and the expense paid by SSDE patients themselves was Chinese Yuan 2627.8 (approximately US$403.9), which were 5.5 and 4.5 times higher than non-SSDE patients (both *P* < 0.001). The annual total expense on Chinese medicine and western medicine were 35.6 times and 78.4% higher in SSDE group than in non-SSDE group (both *P* < 0.001). Moreover, indirect costs associated with the treatment were 70.0% higher in SSDE group. In SSDE group, the score of Zung Self Rating Anxiety Scales had significantly positive correlation with total medical expenditure and the expense on Chinese medicine (ρ = 0.399 and ρ = 0.400,both *P* = 0.019). Nevertheless, total medical expenditure paid by the patients in non-SSDE group positively correlated with the score of Ocular Surface Disease Index (ρ = 0.386, *P* = 0.035).

**Conclusions:**

Medication expenditures and associated costs is an unignorable economic burden to the patients with SSDE. The medical expense had a significantly correlation with clinical severity of SSDE and the patients’ psychological status.

## Background

Dry eye (DE) is a multifactorial disease of the ocular surface characterized by a loss of homeostasis of the tear film, and accompanied by ocular symptoms, in which tear film instability and hyper-osmolarity, ocular surface inflammation and damage, and neurosensory abnormalities play etiological roles [1]. Sjogren’s syndrome (SS) is the leading cause for aqueous tear-deficiency dry eye, with an annual incidence of 6.92 (95% CI: 4.98–8.86) per 100,000 person and an overall prevalence of 60.82 (95% CI: 43.69–77.94) cases per 100,000 [2]. This syndrome exhibits a female-to-male ratio of 9:1 and mainly affects middle-aged women [3], which is characterized by lymphocytic infiltration of exocrine glands and glandular dysfunction, with the salivary and lacrimal glands mainly involved. Compared to patients with non-SSDE, those with SSDE have more severe symptoms and signs, more complicated and challenging treatment, and worse prognosis.

It has been reported that the expenditures and associated costs paid for the treatment of DE are an unignorable economic burden to the patients [4–7], especially after the introduction of cyclosporine emulsion in 2003 [8]. Previous surveys performed in some Asian and European countries showed that the treatment of DE caused an annual cost up to US$1.10million to the healthcare system [5, 6]. Moreover, symptoms of anxiety are common in patients with DE [9, 10]. Anxiety has been shown to have significant association with excessive direct and indirect medical costs [11]. The studies on the expenditures and psychological changes in patients with DE have been performed previously [4–7]. However, no literature has been published regarding the medical cost for SSDE in Asian area. Based on previous findings, we hypothesize that the expenditure and associated costs paid for the treatment of SSDE had a close relationship with its clinical severity and patients’ anxious status. Therefore, we conducted a cross-sectional study to investigate the expenditures associated with the treatment of SSDE, and explored its correlation with the clinical severity and patients’ psychological status as well.

## Methods

### Study population

The study was approved by the ethics committee of Eye & ENT Hospital of Fudan University and was conducted according to the tenets of the Declaration of Helsinki. Patients referring to the dry eye clinic of Eye& ENT Hospital of Fudan University from March to July, 2016 were recruited. Informed consent was obtained from all enrolled patients.

Participants were categorized into two groups: SSDE and non-SSDE. All patients had a frequent or sustained occurrence of any one of DE symptoms for at least 3 months: burning sensation, foreign body sensation, sensation of stabbing pain, gargalesthesia, photophobia, dryness, or asthenopia. For the symptomatic subjects, the diagnosis of DE was made in the case that any two of the following three conditions were present: (i) Schirmer I test (S1T) value of < 10 mm / 5 min, (ii) tear film breakup time (TBUT) of < 10 s and (iii) corneal fluorescein staining (CFS) was noted as positive when CFS score ≥ 1. CFS score was evaluated using the National Eye Institute grid based on grades 0–3 in each of the five quadrants (central, nasal, temporal, superior and inferior cornea) for a total score of 15 [12, 13]. Dry eye patients, who met the diagnostic criteria proposed by the American College of Rheumatology [14, 15], were assigned to SSDE group. The criteria include: (1) positive serum anti-SSA/Ro and/or anti-SSB/La (or positive rheumatoid factor and ANA titer C1:320); (2) labial salivary gland biopsy exhibiting focal lymphocytic sialadenitis with a focus score ≥ 1per4mm^2^; and (3) keratoconjunctivitis sicca with ocular staining score ≥ 3.The other participants were enrolled in the non-SSDE group.

Participants who had the following medical history were excluded: active cornea disorders such as cornea ulcer, corneal infections, tumors and other immunological corneal disorders (e.g. Steven-Johnson syndrome), history of cornea trauma, history of ocular surgery or contact lens wear within the past 6 months, psychiatric disorder with current use of mental medications that might affect the psychological assessment.

### Sequence of examination

Demographic data of eligible subjects were obtained by chart review. The information was collected including name, gender, age, education, occupation, the time when the diagnosis of SSDE/non-SSDE were first made and the duration of disease. Three questionnaires were completed by the patients independently. For those who had difficulty in understanding the questions, the research staff would provide them explanations in a neutral manner. The completed questionnaires were reviewed by research staff to ensure no questions were missing. After demographic data collection and accomplishment of questionnaires, all participants underwent standard ocular examination for DE including CFS, TBUT, and S1T. TBUT and CFS were performed during slitlamp biomicroscopy examination. S1T was performed lastly to avoid the impact on corneal epithelium and fluorescence staining. All ophthalmologic examinations were done by the same professional ophthalmologist.

#### Questionnaire on medical expenditures

The questionnaire, designed by the research group, contained two parts with a total of twelve questions including treatment regimens and the expense paid on the treatment directly or indirectly. Chinese medicine has been confirmed to be an effective method in the treatment of autoimmune disorder and DE by previous studies [16–20]. Therefore, the medical expenditures on western medicine and Chinese medicine were listed and calculated separately. The questions regarding the treatment in the questionnaire were specific for DE and SS. Treatment costs for other diseases were excluded. The detailed content of the questionnaire was listed in Table 1.

**Table 1:** Detailed Content of The Questionnaire on Medical Expense

Category	Questions	Choices and appendix questions
Medical Treatment	Q1.Treatment regimens/option	A:Western medicine B:Chinese medicine C: combination of A and B
	Q2. Western medicine usage: name of medicine, price, frequency of administration.	
	Q3. Chinese medicine usage: name of medicine, price, frequency of administration.	
	Q4. Frequency of medical care	Once a week? Twice a month? Once a month? Once a quarter? Other: please specify
	Q5. Auxiliary therapy or not	If yes, A:moisture chamber glasses B: lacrimal plug implantation C: combination of A and B
Expense on Medical Treatments	Q6. Total expense on medical treatment per month	
	Q7.Expense on western medical treatment per month	
	Q8. Expense on Traditional Chinese medical treatment per month	
	Q9. Indirect costsfor activities associated with medical treatment, such as transportation, nutrition, shelter,and household appliance	
	Q10. The amount of expense paid by yourself (uncovered by insurance)	
	Q11. Your personal income per year	
	Q12. Self-perception of the economic burden	A: barely affected B: affected to some extent but can be accepted C:affected to a large extent

#### Zung self rating anxiety scales (SAS) and ocular surface disease index (OSDI) scale

The Zung Self Rating Anxiety Scales (SAS) was a 20-item, four-point Likert scale self-report assessment for anxiety [21]. Fifteen items expressed negative experience or symptoms (e.g., “I tend to be confusing or terrified”) and 5 expressed positive experience (e.g., “I feel that everything is all right and nothing bad will happen”). Item responses of SAS were ranked from 1 to 4, with higher scores indicating more frequent symptoms. The raw total scores were converted to a 100-point scale by multiplying by 1.25. According to previous study, converted scores> 50 indicated that the patient had the symptoms of anxiety [10]. Additionally, the OSDI scale, a widely used questionnaire to quantify ocular disability in cases of DE [22], was also completed by participants to evaluate the severity of ocular discomfort symptoms.

### Statistical analysis

All data were analyzed with the Statistical Package for the Social Sciences (SPSS, version 18.0, SPSS Inc., Chicago, Illinois, USA). Normal distribution data were expressed as mean ± standard deviation and abnormal distribution data were expressed as median with range. Chi-square test was used to analyze the categorical variables including the gender distribution, income distribution, education, and occupation. Mann-Whitney U tests and Kruskal–Wallis test were applied to compare continuous variables including age, expense, OSDI and SAS scores, CFS, S1T and TBUT. Spearman’s rank correlation test was performed to analyze the relationship between the medical expenditures, SAS scores and clinical parameters. All tests were considered statistically significant at *P* < 0.05.

## Results

A total of sixty-four patients were enrolled in this study, with an average age of 53.7 ± 14.7 years old and females accounting for the majority (85.9%). Thirty-four patients were diagnosed as SSDE, among whom twenty-five (73.5%) were primary SS and nine (26.5%) were secondary to other autoimmune diseases such as rheumatoid arthritis and systemic lupus erythematosus. Another thirty non-SSDE patients served as controls. The demographic data were presented in Table 2. The SSDE group had a lower educational background and a larger number of retired subjects (*P* = 0.020 and 0.016, respectively). Moreover, the time duration from the first onset of the disorder was longer in SSDE patients than non-SSDE ones (6.6 ± 2.4 years vs 1.9 ± 0.4 years) (*P* < 0.001).

**Table 2:** Demographic Characteristics of Study Population

Feature		SSDE group	non-SSDE group	*P* value
		(*n* = 34)	(*n* = 30)	
Age(yrs)	Average	60.8 ± 9.1	57.1 ± 11.2	0.218+
	Range	48–78	39–78	
Gender	Female	31 (91.2%)	24 (80.0%)	0.199#
	Male	3 (8.8%)	6 (20.0%)	
Education	Illiteracy	1 (2.9%)	0 (0.0%)	0.020*#
	Primary school	6 (17.6%)	7 (23.3%)	
	Junior middle school	8 (23.5%)	6 (20.0%)	
	Senior middle school	14 (41.2%)	4 (13.3%)	
	University	3 (8.8%)	11 (36.7%)	
	Post-graduate	2 (5.9%)	2 (6.7%)	
Occupation	Retired	28 (82.4%)	12 (40.0%)	0.016*#
	Unretired	6 (17.6%)	18 (60.0%)	

*SSDE* Sjogren’s syndrome dry eye, + Mann-Whitney U test

*P < 0.05

#Chi-square test

**Fig. 1:**
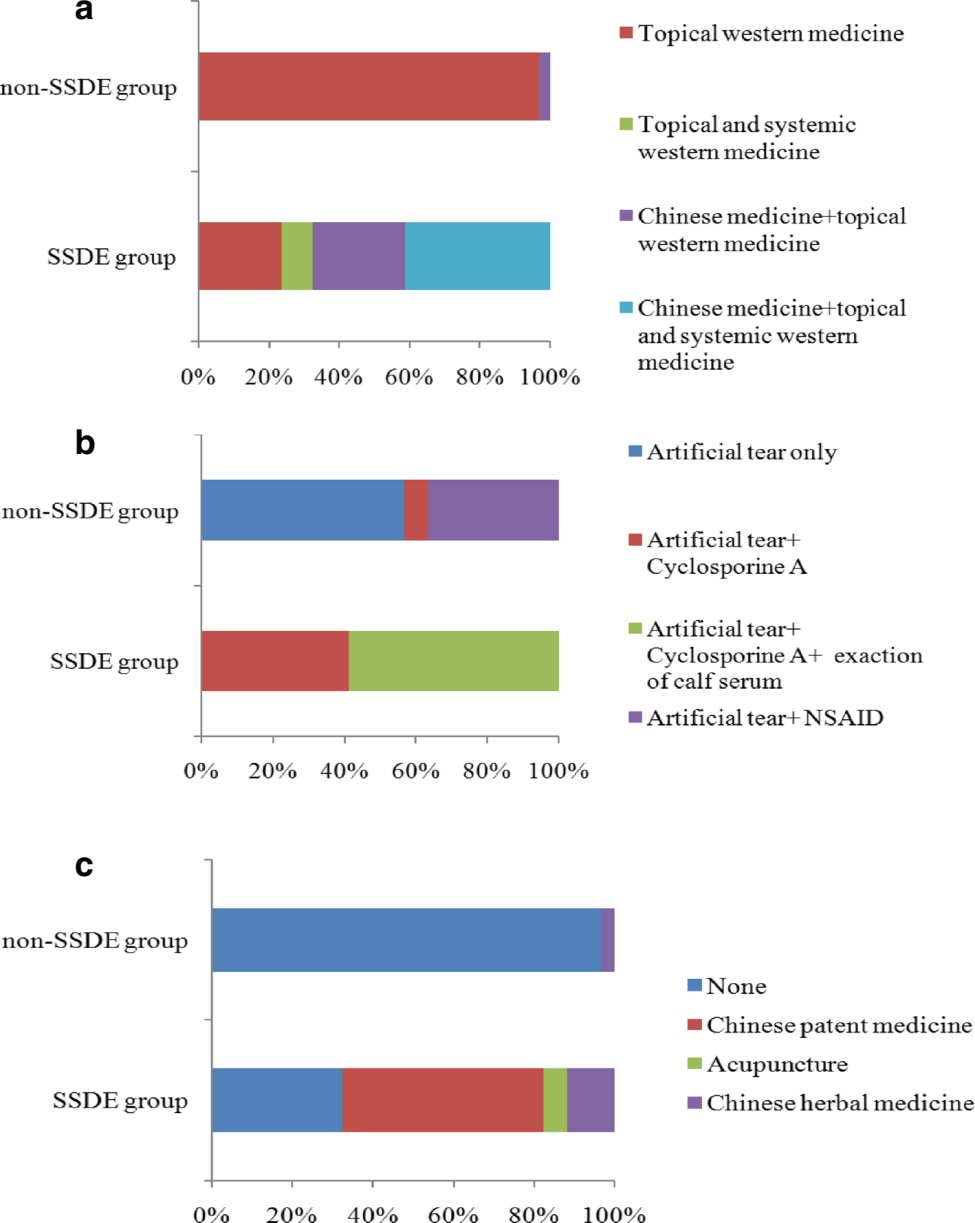
Comparisons on the therapeutic regimens between SSDE and non-SSDE group. **a** showed the combination of treatment and medication. Eighteen (52.9%) SSDE patients had oral systemic western medications including hydroxychloroquine and/or corticosteroid. Twenty-five (73.5%) SSDE patients and 1 (3.3%) non-SSDE patient took combined treatment of western and traditional Chinese medicine. **b** illustrated the therapeutic regimen of western medicine. Artificial tear was used by dry eye patients in both two groups. However, 100% of SSDE patients had topical administration of cyclosporine A, while only 6.7% of non-SSDE subjects had the treatment of cyclosporine A. Moreover, 58.8% of SSDE patients had the treatment of calf serum extraction, whereas none of non-SSDE patients had this treatment. **c** presented the details of treatment by Chinese medicine. 50% of SSDE subjects took Chinese patent medicine. Meanwhile, 5.8% and 11.8% of SSDE subjects used acupuncture and Chinese herbal respectively

Figure 1a showed that eighteen (52.9%) SSDE patients had oral systemic western medications including hydroxychloroquine and/or corticosteroid, whereas none of patients in non-SSDE group had systemic treatment of western medications. Moreover, a larger proportion of patients in SSDE group (73.5%) had combined treatment of western medicine and traditional Chinese medicine than in non-SSDE group (3.3%). The therapeutic regimens were more complicated and diversified in the SSDE group. All SSDE patients had topical administration of cyclosporine eye drops, while only 6.7% of non-SSDE subjects had the same treatment. The detailed information of topical medication usage was shown in Fig. 1b and c. It’s also notable that the SSDE patients referred to the dry eye clinic more positively and regularly. In our study, 32 (94.1%) SSDE patients referred to our hospital once a month. On the contrary, the interval time of medical visit was irregular in the non-SSDE group, ranging from one month to six months.

The annual total expenditure on medical treatment in the SSDE group was 5.5 times higher than in the non-SSDE group. Annual costs on Chinese medicine in the SSDE group were 35.6 times higher than the non-SSDE group, accounting for a significant portion of total expenditure in the medical treatment. The annual medical expense on western medicine was also 78.4% higher in SSDE patients than in non-SSDE ones, which were shown in Table 3.

**Table 3:** Medical Expenses and Detailed Costs for Each Part

			SSDE group	Non-SSDE group	*P* value
Annual Medication Expense(CNY)	Total	Range	780.0–24,537.6	720.0–6256.0	
		Average	7637.2 ± 6079.0	1179.1 ± 990.4	< 0.001‡
		Median	6505.9	900.0	
	Western medicine	Range	780.0–2937.6	576.0–1680.0	
		Average	1774.9 ± 596.2	995.0 ± 267.8	< 0.001‡
		Median	1839.2	900.0	
	Chinese medicine	Range	0–21,600.0	4800.0	
		Average	5862.4 ± 5706.3	NA	< 0.001‡
		Median	4620.0	NA	
Annual Medication expense paid by Patients Themselves (Uncovered by Insurance) (CNY)	Total	Range	297.5–7896.2	271.2–2220	
		Average	2627.8 ± 1857.0	481.9 ± 393.3	< 0.001‡
		Median	2243.04	338.9	
	Western medicine	Range	289.0–1800.0	271.2–780.0	
		Average	750.2 ± 288.4	405.8 ± 151.7	< 0.001‡
		Median	741.5	338.9	
	Chinese medicine	Range	0–6720	1560	
		Average	1845.5 ± 1768.9	NA	< 0.001‡
		Median	1500.0	NA	
Annual indirect expense (CNY)		Range	96–8400	96–8124	
		Average	828.0 ± 1866.0	487.2 ± 1404.0	0.017 *
		Median	228	96	
Expense on auxiliary therapy paid by users (CNY)		Range	300–6000	400	
		Average	2757.1 ± 2496.0	NA	
		Median	1750	NA	

*CNY* Chinese Yuan, *SSDE* Sjogren’s syndrome dry eye, *NA* not applicable

The range of expense was listed in the brackets

*P < 0.05

‡ *P* < 0.001

*P* value by Mann-Whitney U test

All enrolled patients had the same type of medical insurance, which is provided by local government and is mandatory to all citizens. Medical expense on the treatment of dry eye was predominantly paid by insurance both in the SSDE group (94.1%) and in the non-SSDE group (80.0%). Even though, the expenditures uncovered by medical insurance were significantly higher in the SSDE group. The costs paid by SSDE patients themselves on western medicine and Chinese medicine were 84.9% and 34.5 times higher than non-SSDE ones respectively, as shown in Table 3. It’s noteworthy that indirect expenditures and expenses on auxiliary therapy were also significantly higher in the SSDE group (*P* = 0.017 and *P* < 0.001). There was a higher proportion of SSDE subjects taking auxiliary therapy than non-SSDE ones (20.6% vs 3.3%, *P* < 0.001). These costs were not covered by medical insurance, too. In contrast, the personal income is lower in SSDE subjects. Therefore, a significantly higher number of patients in SSDE group considered medical expenditure as a financial burn, as shown in Table 4.

**Table 4:** Personal Income and Self-perception of Economic Burden

		SSDE group	Non-SSDE group	*P* value
Annual Income (CNY)	Range	1400–30,000	2000–20,000	0.001†+
	Average	4494.1 ± 5138.0	6833.3 ± 4077.6	
	Median	3000	6000	
Annual Income distribution	≤3000CNY	25(73.5%)	9(30%)	0.002†#
	3000-10000CNY	6(17.6%)	12(40%)	
	≥10,000 CNY	3(8.9%)	9(30%)	
Self-perception of economic burden	Uninfluenced	5(14.7%)	27(90.0%)	< 0.001‡#
	Limited economic burden but acceptable	9(55.9%)	3(10.0%)	
	Heavy economic burden	10(29.4%)	0(0.0%)	

*CNY* Chinese Yuan, *SSDE* sjogren’s syndrome dry eye

† *P* < 0.01

‡ *P* < 0.001

+ Mann-Whitney U test

#Chi-square test

The ophthalmologic and psychological data were presented in Table 5. Mean CFS score was significantly higher in the SSDE group (*P* < 0.001), and the positive rate of CFS as well (83.8% vs 46.7%, P < 0.001). Among SSDE patients with CFS staining, 14.7% were unilaterally affected and 79.4% were bilaterally affected. Moreover, SSDE patients reported a higher OSDI score and SAS score (both *P* < 0.001). 26.5% of SSDE patients (*n* = 9) had the symptoms of anxiety, while only 6.7% of non-SSDE patients (*n* = 2) tended to be anxious (*P* = 0.036).

**Table 5:** Ophthalmologicaland psychological data in both groups

	non-SSDE group		SSDE group		*P* value	
	OD	OS	OD	OS	OD	OS
TBUT	3.1 ± 1.1	3.0 ± 1.5	3.2 ± 1.3	3.4 ± 1.2	0.681	0.054
S1T	9.0 ± 7.1	10.4 ± 8.8	6.0 ± 3.0	6.2 ± 3.1	0.176	0.147
CFS	0.9 ± 1.1	0.7 ± 1.1	3.2 ± 2.7	3.6 ± 2.7	< 0.001‡	< 0.001‡
OSDI	30.0 ± 7.9		51.1 ± 20.2		< 0.001‡	
SAS	34.7 ± 8.0		44.0 ± 9.2		< 0.001‡	

*TBUT* tear film breakup time, *S1T* Schirmer 1 test, *CFS* corneal fluorescein staining, *OSDI* ocular surface disease index, *SAS* self-rating anxiety scale, *SSDE* Sjogren syndrome dry eye

Values are presented as the mean ± standard deviation. P value by Mann-Whitney U test

‡ *P* < 0.001

The spearman’s correlation analysis revealed that SAS scores had significantly positive correlation with both total medical expenditure and expense on Chinese medicine in the SSDE group (ρ = 0.399 and ρ = 0.400,both *P* = 0.019). Similar correlations were also found between SAS scores and the costs paid by the patients themselves on total medication and Chinese medicine (ρ = 0.417 and ρ = 0.407, *P* = 0.014 and *P* = 0.017). SAS scores also had significant correlation with OSDI scores, education and S1T values in SSDE patients (ρ = 0.445, − 0.375 and − 0.416, *P* = 0.008, 0.029 and 0.014, respectively). In contrast, ODSI scores were found to positively correlate with the costs paid by the patients themselves on total medication and western medicine in non-SSDE group (ρ = 0.386 and ρ = 0.677, *P* = 0.035 and *P* < 0.001). Western medicine expenditures had no significant correlation with SAS and clinical parameters in both groups. What’s more, no correlation was found between the time from onset of disease and SAS score in all patients.

## Discussion

The current study showed that the annual medical costs for treatment of SSDE was CNY 7637.2 (approximately US$1173.8) on average, which was similar to those reported in the United Kingdom [6]. It far exceeded the per capita health care expense in Shanghai, which was reported to be CNY1016.7 (approximately US$146.774) for an urban family and CNY1029.0 (approximately US$166.77) for a rural family [23]. Based on the per capita total income of both urban and rural households reported by the same website, the annual medical expense paid by the patients themselves on the treatment of SSDE accounted for 5.9% and 14.8% of the per capita total income for urban and rural households respectively. In contrast, the annual medical expense uncovered by insurance was CNY 481.9 (approximately US$71.1) in the non-SSDE group, which only accounted for 1.1% and 2.7% of urban and rural household annual income. Therefore, the medical expenditures accounted for a considerably higher proportion of per capita total income in the SSDE group than in the non-SSDE group both in urban and rural families, especially in rural families.

Compared to non-SSDE patients, SSDE patients suffered from more severe clinical symptoms and signs, and had stronger motivation for medical help and more regular medical visit. Meanwhile, the therapeutic regimens to treat SSDE were more complicated, including artificial tears, topical applications of immunosuppressant therapies, autologous serum or serous extraction, secretagogues and interventional treatments, and had more diversified combinations [24]. Some of them were not or only partially covered by medical insurance. The current study found that even with medical insurance, medical expense paid by the patients themselves and associated expense was significantly higher in the SSDE group than that in the non-SSDE group. According to our study, the annual burden of the SSDE and non-SSDE for the Chinese healthcare system was estimated to be CNY 5009.4.1(approximatelyUS$769.9) and CNY697.2 (approximatelyUS$102.4) per patient. Medical expense on the treatment of SSDE leads to a heavy economic burden on both patients themselves and healthcare system.

The main reason for higher expenditure on western medicine in SSDE patients was that cyclosporine ophthalmic solution, which was indispensable in the treatment of SSDE, was uncovered by medical insurance in Shanghai. Nevertheless, the exaction of calf serum (Deproteinized Calf Blood Extract Eye Drops, Xingqi Co. Ltd.) and artificial tears were only partially covered by medical insurance. Unlike non-SSDE patients who usually use artificial tears only, cyclosporine eye drops and the serum exaction were necessary in the treatment of SSDE to inhibit ocular surface inflammation, promote corneal epithelium healing and relieve ocular discomfort symptoms [25–27]. Moreover, SSDE patients usually used medications more regularly and frequently, leading to a larger demand for the medications and higher medical expenditures.

Many studies had been confirming that Chinese medicine, including herbal medicine, acupuncture, moxibustion and cupping, was an effective method in the treatment of SS and DE [16–20]. Our study showed that the expenditure on Chinese medicine treatment accounted for 80.3% of total medical costs in the SSDE group, being significantly higher than that in control group. Acupuncture, which had been proven to be effective in stimulating tear production [20], was not fully covered by medical insurance. Continuous price rising of Chinese herbal medicine in the recent year and partial coverage or uncoverage of some precious and expensive Chinese herbs by medical insurance system were also the contributing factors for higher medical costs in SSDE group.

Apart from direct expense on medical treatment, SSDE patients also had more indirect expenditure. The majority of SSDE patients received combined treatment with western medicine and traditional Chinese medicine at the same time. To save time and accomplish clinical visits to several hospitals in one day, SSDE patients are likely to spend more costs on transportation, food or even accommodation. Some severe SSDE patients made the extra expense to buy household electrical appliance, for instance air humidifier, to make the household environment more comfortable and to provide extra help for the relief of dry eye symptoms. Moreover, auxiliary therapies including wearing moisture chamber glasses and lacrimal plug implantation need a large amount of money. Unfortunately, these costs were not covered by medical insurance, too. Higher proportion of subjects using auxiliary therapy also contributed to the higher medical expenditure in the SSDE group.

The current study found that SSDE patients showed more signs of anxiety than non-SSDE patients, which was in agreement with previous reports [10, 28]. Apart from more severe ocular symptoms and clinical signs, heavier economic burden was another important reason for more severe anxiety in SSDE patients. More anxiety and depression lead to deterioration of ocular symptoms and further more expenditure on medical treatment, which formed a vicious cycle. A potential mutual promotion relationship between psychological status and medical expenditure might be the supporting evidence [11, 29]. Additionally, some SSDE patients were secondary to other autoimmune disorders. Systematic symptoms might be another potential contributing factor aggravating the anxiety and depression.

In our study, medical expenditures and Chinese medicine costs, no matter total expenses or the amount paid by the patients themselves, were found to be positively correlated with SAS scores in the SSDE group. In contrast, the expenses paid by the patients themselves on total medication and western medicines were found to correlate with ODSI scores in the non-SSDE group. The possible reason was that the majority of SSDE patients had a lower educational background and had the retirement in their early fifties. With limited amount of retirement income, the cost for medical treatment was a heavier economic burn to them compared to the non-SSDE ones. Economic burden and life pressure were far more likely to induce anxiety in SSDE patients. On the contrary, non-SSDE patients were relatively better educated and had higher income. The medical expenditure on dry eye can hardly induce anxiety in this population. The leading factor for their medical cost was their subjective feeling of ocular discomfort. In summary, In regard to mild to moderate dry eye patients, clinical signs and symptoms are still the important index from the doctor’s perspective. As for SSDE patients, more care should be paid to their psychological status during the treatment.

The present study revealed a positive correlation between SAS scores and OSDI scores in both SSDE and non-SSDE groups, which was in agreement with Li’s study [10]. Moreover, SAS scores had negative correlation with S1T, which was not shown before. Since SS was the most common cause for tear-deficiency dry eye, S1T test might be more meaningful with regard to the diagnosis of SSDE. In addition, many studies confirmed a significantly negative correlation between anxiety and educational level [10, 30]. It was in agreement with our findings. More medical information through various paths and a better understanding of the disease is of great importance to improve SSDE patients’ cooperation with the doctors and alleviate their symptoms of ocular discomfort and anxiety effectively.

There were three limitations in this study. First, the sample size is not big enough. Further study with a larger sample size is required to confirm these findings. Second, the annual medical expenditures were calculated mainly based on the medical costs in the several recent months because most patients could not recall the expense occurring a long time ago. Considering the price fluctuation of medicine and modification of treatment regimen, the biases in calculating the medical expense were unavoidable. The medical expense might be over-estimated in the patients with deteriorated symptoms in the recent months, while it might be under-estimated in those with improvements in the recent months. Third, only SAS scale was used to evaluate the anxiety of the patients. There were some other psychological scales, such as Self Rating Depression Scales (SDS) [31], which might be used in our further study to merit overall evaluation of the psychological status.

## Conclusions

Medical expense is a considerable economic burden to patients with SSDE in East China. The medical expense had a significant correlation with psychological status in SSDE patients. Apart from the improvement of clinical symptoms and signs, attentions should also be paid to alleviate the anxiety caused by the disease. It is also necessary to promote public education to SSDE patients and help them to get a better understanding of the disease. In addition, effective and inexpensive treatment is urgently needed to reduce the financial burden of SSDE on both patients and healthcare systems.

## Abbreviations

CFS: Corneal fluorescein staining
DE: Dye eye
OSDI: Ocular Surface Disease Index
S1T: Schirmer I test
SAS: Zung Self Rating Anxiety Scales
SDS: Self Rating Depression Scales
SS: Sjogren’s syndrome
SSDE: Sjogren’s syndrome dry eye
TBUT: Tear film breakup time
